# Decellularized Extracellular Matrix Scaffolds for Cardiovascular Tissue Engineering: Current Techniques and Challenges

**DOI:** 10.3390/ijms232113040

**Published:** 2022-10-27

**Authors:** Greta Ionela Barbulescu, Florina Maria Bojin, Valentin Laurentiu Ordodi, Iacob Daniel Goje, Andreea Severina Barbulescu, Virgil Paunescu

**Affiliations:** 1 Immuno-Physiology and Biotechnologies Center (CIFBIOTEH), Department of Functional Sciences, “Victor Babes” University of Medicine and Pharmacy, No 2 Eftimie Murgu Square, 300041 Timisoara, Romania; 2 Department of Clinical Practical Skills, “Victor Babes” University of Medicine and Pharmacy, No 2 Eftimie Murgu Square, 300041 Timisoara, Romania; 3 Clinical Emergency County Hospital “Pius Brinzeu” Timisoara, Center for Gene and Cellular Therapies in the Treatment of Cancer Timisoara-OncoGen, No 156 Liviu Rebreanu, 300723 Timisoara, Romania; 4 Faculty of Industrial Chemistry and Environmental Engineering, “Politehnica” University Timisoara, No 2 Victoriei Square, 300006 Timisoara, Romania; 5 Department of Medical Semiology I, “Victor Babes” University of Medicine and Pharmacy, No 2 Eftimie Murgu Square, 300041 Timisoara, Romania; 6 Advanced Cardiology and Hemostaseology Research Center, “Victor Babes” University of Medicine and Pharmacy, No 2 Eftimie Murgu Square, 300041 Timisoara, Romania; 7 Center for Advanced Research in Gastroenterology and Hepatology, Department of Internal Medicine II, Division of Gastroenterology and Hepatology, “Victor Babes” University of Medicine and Pharmacy, 300041 Timisoara, Romania

**Keywords:** decellularization, recellularization, 3D scaffold, extracellular matrix, tissue engineering, regenerative medicine

## Abstract

Cardiovascular diseases are the leading cause of global mortality. Over the past two decades, researchers have tried to provide novel solutions for end-stage heart failure to address cardiac transplantation hurdles such as donor organ shortage, chronic rejection, and life-long immunosuppression. Cardiac decellularized extracellular matrix (dECM) has been widely explored as a promising approach in tissue-regenerative medicine because of its remarkable similarity to the original tissue. Optimized decellularization protocols combining physical, chemical, and enzymatic agents have been developed to obtain the perfect balance between cell removal, ECM composition, and function maintenance. However, proper assessment of decellularized tissue composition is still needed before clinical translation. Recellularizing the acellular scaffold with organ-specific cells and evaluating the extent of cardiomyocyte repopulation is also challenging. This review aims to discuss the existing literature on decellularized cardiac scaffolds, especially on the advantages and methods of preparation, pointing out areas for improvement. Finally, an overview of the state of research regarding the application of cardiac dECM and future challenges in bioengineering a human heart suitable for transplantation is provided.

## 1. Introduction

Cardiac transplantation is the definitive treatment for end-stage heart failure. Patients with this condition require frequent interventions and hospitalizations, encountering a poor quality of life. Medical and device therapy can be offered temporarily to patients with end-stage heart failure, but it does not replace cardiac allotransplantation [1]. There are some critical limitations of heart transplantation for which researchers are constantly trying to find solutions. First of all, the chronic shortage of donor organs is a major hurdle to this approach. The number of patients on a waiting list for a heart transplant increases each year faster than the number of donors. This unfortunate situation has been seen in the US as well as in Europe [2,3]. Secondly, the life-long immunosuppression required post-transplantation and keeping the balance between rejection and infection have a significant impact on the long-term survival rate [4,5].

Cardiac tissue engineering holds promise to solve various heart diseases and the organ donor shortage problem. The field of tissue engineering involves the development of biological substitutes that can restore, improve or maintain tissue function. The goal is to develop a scaffold that can mimic the characteristics of a native healthy myocardium. This outcome allows the engineered tissue to deliver and enhance the survival and differentiation of cardiovascular cells. Although various synthetic materials have been studied as scaffolds, they have not been able to mimic the complex architecture and function of the native heart [6].

This exciting field in regenerative medicine has made significant advances in understanding various aspects of the human heart. Most cardiovascular tissue engineering work has been accomplished on vascular and valve disease, congenital heart disease, and, more recently, heart failure and ischemic heart disease [7,8,9,10].

The ultimate goal of regenerative medicine is to create bioartificial hearts with personalized cells that make the concept of autologous tissue engineering conceivable. The first attempt to build a bioartificial heart was pioneered by Ott et al. in 2008. The researchers used a decellularized rat heart extracellular matrix (ECM) as a support network for neonatal rat cardiomyocytes (CMs) and rat endothelial cells to connect and create a whole recellularized construct [11].

**Figure 1:**
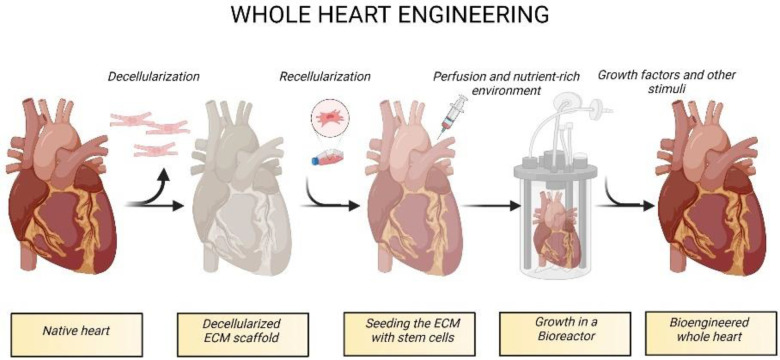
Growing heart in a bioreactor. Building a heart starts with the decellularization of the organ, which creates an acellular scaffold consisting of structural proteins such as collagen and laminins, as well as proteoglycans and polysaccharides. This scaffold is further seeded with cells and cultured in a bioreactor to mimic the natural heart functions. A bioreactor supports and protects the engineered construct, providing nutrients and a sterile environment. This technique would create a functional bioartificial heart, a theoretical alternative to transplantation. Created with BioRender.com.

The generation of functional bioengineered organs is very complex. The procedure comprises two essential steps (Figure 1). The initial step is called decellularization and creates a naturally derived three-dimensional (3D) ECM by removing all cells from an animal or human organ [12]. One of the most critical aspects of the decellularization process is to ensure that the ECM is well-preserved and that it can support the growth of new cells [13]. Second, these acellular scaffolds need to be recellularized. Recellularization is defined as the repopulation of an acellular scaffold with organ-specific cells, recreating its function. Although many ECM-like materials have been used for engineering organs, the maturation and rearrangement of cells are best guided by acellular biological scaffolds [14].

Since the concept of decellularization was first proposed, numerous studies have been carried out on the various aspects of this process. The timeline of significant milestones using decellularized ECM (dECM) scaffolds for myocardial repair started with developing an acellular rat heart by antegrade coronary perfusion on a Langendorff apparatus [11]. In recent years, the concept of whole-organ decellularization has been extended to larger hearts. Wainwright and his team described for the first time a reproducible decellularization technique of the whole porcine heart. Serial perfusion with acidic and enzymatic solutions was used for less than ten hours to remove cellular elements. Immunohistochemistry demonstrated the absence of nuclei and muscle cells after decellularization while preserving collagen and elastin, as well as mechanical integrity [15]. In the subsequent years, perfusion-based decellularization protocols were used to obtain human-size cardiac scaffolds [16,17,18]. Sanchez et al. obtained the first acellular human whole heart scaffold, which preserved 3D architecture, vascular integrity, and chamber geometry [19].

Aside from producing acellular organs, decellularization has also been used to create cardiac dECM slices. Animal or human left ventricular myocardium was sectioned into thick layers followed by decellularization protocols. Several studies have been performed on cardiac dECM patches’ properties, including their cell-matrix adhesion, proliferation, and differentiation using stem cells, such as embryonic (ESCs), mesenchymal (MSCs), and induced pluripotent stem cells (iPSCs) [20,21,22].

This review will focus on dECM in cardiac tissue engineering, with variations in tissue and species sourcing. Due to the varying sizes of the decellularized heart, these differences could affect the development of effective protocols for human use. We will specifically provide an overview of the techniques used to obtain the dECM and discuss its great potential as a tissue engineering platform.

## 2. Composition and Function of Cardiac dECM

The extracellular matrix is a non-cellular 3D macromolecular network, an essential natural tissue component. It comprises three major elements: glycosaminoglycans (GAGs), adhesive glycoproteins, and fibrous proteins that provide tensile strength (e.g., collagen, elastin). ECM supports cell adhesion and helps form various cell behaviors, such as differentiation and migration. It is demonstrated that dECM provides a dynamic microenvironment that dictates the stem-cell fate through transmembrane receptors (e.g., integrins). Evidence sustains the idea that ECM can trigger essential biological activities for normal tissue development. Modifications in ECM structure seem to be associated with various diseases [23,24].

ECM can be structured in two main regions: the basement membrane and the interstitial matrix. The basement membrane promotes cell polarity and functions such as migration and differentiation via cell surface receptors [25]. The second ECM compartment provides mechanical and structural support to the tissue and mainly comprises collagen I and III molecules [26].

ECM provides a specific organizational microenvironment for cell mitosis and morphogenesis [27]. Decellularized scaffolds are a promising carrier for stem cells in tissue engineering because of their remarkable similarity to the original tissue [28]. For instance, rat liver acellular scaffolds were used as ECM to lead the differentiation of stem cells to hepatocytes. After several days in culture, the cells lost the embryonic markers and expressed hepatic genes encoding cytochrome P450s and albumin [29].

The complex architecture of the cardiac structure makes it nearly impossible to recapitulate de novo. The macro and microstructure vary with each cardiac chamber and valve [30]. Although 3D printing and biomaterial chemistry are commonly used to create solid organs, it remains a challenge because of the complex cell-laden structures required [31]. Alternatively, researchers have focused on creating acellular cardiac scaffolds from xenogeneic sources or organs unsuitable for transplantation.

Decellularized human hearts using standardized protocols show the preservation of the four largest ECM-protein families (collagens, fibrillins, laminins, and proteoglycans). Further analysis confirms cardiac matrix architecture and composition maintenance. Decellularized vasculature maintains structural blood vessel characteristics, with intact vascular hierarchy and no basement membrane disruption [32].

There are no negative effects on the anisotropic behavior of the human decellularized myocardium on passive mechanical testing. Laser-cut decellularized myocardium exhibits stronger peak stresses and faster twitch kinetics than the electrospun gelatin-based scaffolds. These findings suggest that decellularized cardiac ECM offers in vitro tissue formation advantages over the synthetic framework [33].

Studies show that acellular cardiac ECM scaffolds can trigger endogenous tissue repair mechanisms. A dynamic interchange between the acellular ECM and the host cells is essential to tissue development and wound healing [34]. A study by Svystonyuk et al. proved that acellular bioscaffolds used in rodent model myocardial injury redirect cardiac fibroblasts, reducing fibrosis and stimulating new blood vessel formation. The preclinical observations were translated to humans with ischemic heart injury proving that human cardiac fibroblasts on intact bioscaffolds downregulate fibrotic genes and upregulate vasculogenic genes [35].

Due to the complexity and physical properties, the composition of the acellular cardiac scaffolds is challenging to identify and quantify [36]. Using various detergent treatments, Liguori et al. decellularized different cardiovascular tissues (left ventricle, mitral valve, and aorta) and determined the protein composition using mass spectrometry analyses. All three tissues contained collagens as their main ECM component, predominant collagen VI for the left ventricle and mitral valve, and elastin for the aorta [37]. Johnson et al. decellularized six human cadaveric hearts to understand human myocardial ECM protein composition at a quantitative level. Results showed significant patient-to-patient variability (e.g., different percentages of fibrillar collagen, basement membrane proteins, structural ECM proteins, and matricellular proteins) [38]. These findings are essential because altering local concentrations of basement membrane collagens and laminins were demonstrated to modulate cardiomyocyte behavior [39]. The compositional differences within the ECM can modulate successful implantation and host immune response [40]. In addition to the essential role of dECM bioactivity in cardiac repair, researchers believe that future studies investigating dECM composition will help complement the understanding of cardiac dECM therapeutic outcomes and expand the clinical applications [41].

Although not yet optimal, the cardiac acellular bioscaffolds approach to functional tissue repair or replacement seems a compelling alternative.

## 3. Key Materials for Cardiac Tissue Engineering

### 3.1. Cell Sources

The primary cell sources in tissue engineering are embryonic stem cells (ESCs), fetal stem cells, adult stem cells, and induced pluripotent stem cells (iPSCs). Each category has its drawbacks, such as ethical and legal issues, limited differentiation capacity, and tumorigenic risk. Differentiation of stem cells depends on the architectural characteristics of the acellular scaffold, cell cultivation and differentiation media, and mechanical/electrical stimulation [42]. Researchers genetically reprogrammed somatic cells into iPSCs using four transcription factors: c-Myc, Klf4, Sox2, and Oct3/4 [43], respectively, Sox2, Oct4, Lin28, and Nanog [44]. Since then, iPSCs have emerged as a key component of cardiac tissue engineering. Different protocols have been developed to obtain the four primary specialized cells of the mammalian adult heart (cardiomyocytes, CMs; endothelial cells, ECs; vascular smooth muscle cells, vSMCs, and cardiac fibroblasts, CFs) [45]. The most common protocol to obtain iPSC-CMs is modulation of the Wnt pathway by adding small molecules (e.g., GSK3 inhibitor CHIR99201) [46]. Some researchers encourage applying adult stem cells in regenerative medicine because they proved lower immunogenicity than iPSCs [47]. Among adult stem cells, adipose stem cells (ASCs) have been widely studied for cell therapies and regenerative medicine. Multiple experiments using animal models demonstrated the ability of ASCs to repopulate acellular scaffolds, improve vascularization of the infarcted area and prevent cell death [48,49,50]. One of the significant challenges remains identifying and selecting the best-suited stem cell type before translating stem cell use from preclinical to clinical studies.

### 3.2. Naturally Derived Scaffolds

**Figure 2:**
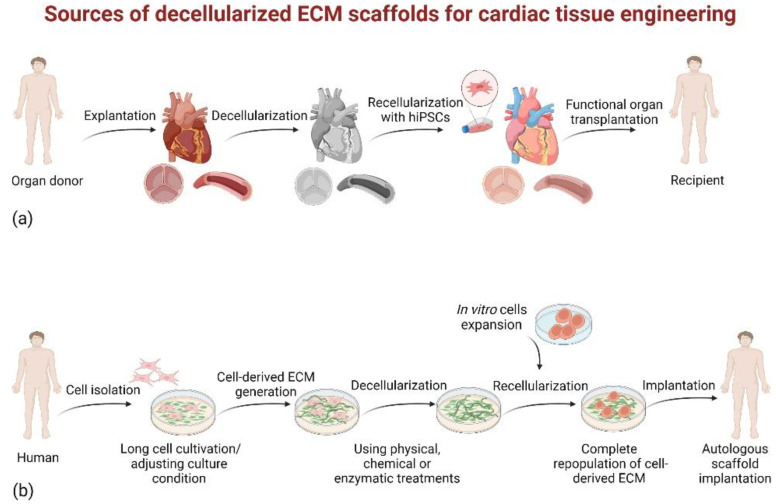
Classification of dECM scaffolds. (**a**) Native tissue-derived dECM scaffolds for tissue engineering. The organ (e.g., porcine heart, cadaveric heart) is harvested from the donor and undergoes the process of decellularization. This step involves the removal of the cellular components, leaving only the ECM, which maintains the organ’s composition, architecture, and mechanical properties. Then, the decellularized scaffold is repopulated with progenitor cells. The recellularized construct can be transplanted into patients after extensive evaluation of its functionality. (**b**) Cultured cell-derived dECM scaffolds for tissue engineering. Cells from different tissues innately generate matrices that mimic the relative composition of the natural tissue ECM. Once sufficient ECM has been deposited, the cellular component can be removed through a combination of decellularization treatments. Subsequently, cells from other sources are reseeded onto the dECM scaffolds to generate bioengineered grafts. Created with BioRender.com.

Cardiac tissue-derived dECM presents many advantages due to organ composition and structure preservation. After proper decellularization, cardiac dECM maintains a highly organized network with promising clinical applications [51,52]. Decellularized cardiac scaffolds can be classified into two categories according to the sources of ECM: native tissue-derived and cultured cell-derived dECM scaffolds [53] (Figure 2).

#### 3.2.1. Native Tissue-Derived dECM Scaffolds

Native tissue-derived dECM scaffolds are a complex assembly of macromolecules obtained from specific organs or tissues. Cardiac scaffolds are used as frameworks along with integrated specialized cells to generate tissue-engineered grafts [54].

Since the first attempt to build a bioartificial heart, researchers have tried different protocols to obtain a decellularized 3D ultrastructure and to simulate cardiac physiology [55]. Investigated decellularization methods include physical, enzymatic, and chemical treatments. Studies showed that combined treatments allow more effective decellularization [56,57].

The concept of tissue-engineered valves, grafts, and whole decellularized hearts has demonstrated promising early results. The main advantage of allografts and xenografts is not requiring lifetime treatment with anticoagulants like artificial valves [58].

Acellular cardiac patches have shown therapeutic outcomes in cardiac injury due to their biomimetic nature. In 2018 Shah et al. used decellularized porcine myocardium as a cardiac patch for adipose-derived stem cells (ASCs) in a rat myocardial infarction model, demonstrating great engraftment on the host tissue [59].

Large animal and human hearts have been decellularized as the perfect source of bioartificial ECM. The porcine heart is often considered suitable for creating ECM scaffolds because it resembles the human heart. However, there are some limitations, such as a possible immune response and transmission of viruses [60,61].

#### 3.2.2. Cultured Cell-Derived dECM Scaffolds

Studies have shown that cultured cell-derived ECM can address some of tissue-derived ECM’s limitations for clinical application (e.g., pathogen transfer, immune response, lack of donors). Cells cultured in vitro secrete cell-specific ECM, which can be decellularized to an excellent acellular scaffold. Some advantages of cell-derived ECM over native tissue-derived ECM are the possibility for large-scale in vitro study and ease of obtaining models of small tissue regions [28].

Sharma et al. described a protocol for generating ECM scaffolds by decellularizing human dermal fibroblasts (hDFs) cell sheets [62]. Cell-derived ECM allows selecting appropriate ECM-producing cell types, genetically modifying, and exposing them to specific stimuli to create an acellular scaffold with desired properties [63].

Numerous applications have been studied for cell-derived ECM, especially in regenerative medicine and tissue engineering. Cell-derived ECM has been explored to engineer heart valves. In 2013 Weber et al. obtained a heart valve prototype from human vascular-derived fibroblast ECM and implanted it in a non-human primate model into the orthotopic pulmonary valve position. The in vivo functionality was surveyed using echocardiography with no significant regurgitation after eight weeks. Microscopic analysis of the explanted valves showed collagen presence and a remarkable homogeneous cellular repopulation [64].

Cell-derived ECM has also been explored to engineer cardiac patches into mouse myocardial infarction models. The acellular scaffold was manufactured from cardiac fibroblasts (CFs) and decellularized with peracetic acid (PAA). Subsequently, recellularization with human embryonic stem cell-derived mesenchymal stromal cells (hEMSCs) provided a cardiac-derived matrix protein composition homologous to the recipient myocardium [65].

The differences between native tissue-derived dECM and cultured cell-derived dECM are reported below (Table 1).

### 3.3. Signals

Cardiac dECM without adding cells or molecular therapeutics has elicited functional improvements in left ventricular ejection fraction (LVEF) and prevention of left ventricular dilation in animal models [66]. However, combining acellular scaffolds with growth factors, cytokines, or other bioactive molecules shows excellent promise as a therapeutic approach. The basic-fibroblast growth factor (bFGF) and vascular endothelial growth factor (VEGF) are the common exogenous pro-angiogenic factors used in cardiac patches. Rajabi et al. created “humanized rat hearts” by seeding human ESC-derived cardiac progenitor cells (CPCs) into the decellularized scaffolds. Perfusion of bFGF improved the retention of CPCs and differentiation into cardiomyocytes, smooth muscle cells, and endothelial cells [67]. Marinval et al. coated decellularized porcine valves with a fucoidan/VEGF polyelectrolyte multilayer film. Results showed that fucoidan/VEGF enhanced human umbilical vein endothelial cells (HUVEC) adhesion, density, and viability [68].

Bioreactor devices can simulate biological and biochemical processes in cardiac tissue engineering to provide controlled and prespecified environmental conditions [42]. Biochemical cues such as oxygen levels, pH, CO_2_ concentration, and nutrients are monitored and maintained at physiological levels using bioreactor-based systems [69]. Bioreactors should provide oxygen at the same rate as being consumed. Cells exposed to hypoxic conditions undergo apoptosis. Carrier et al. investigated the relationship between oxygen concentration (pO_2_) and the composition and metabolic function of engineered cardiac tissue. Results showed that constructs cultured at pO_2_ of 160 mmHg had higher DNA and protein contents, better expression of sarcomeric α-actin, and better contraction rate than constructs cultured at pO_2_ of 60 mmHg [70]. Additionally, studies showed that mechanical stimulation influences the seeded scaffolds and offers dynamic stimuli for the pre-conditioning of cardiac tissue-engineered constructs in vitro [71]. Passive stimulation (e.g., stiffness, topography, static stress) and active stimulation (e.g., cyclic strain, compression, perfusion) mimic mechanical forces that the cardiac tissue undergoes over development [72]. Exogenous electrical stimulation is known to affect cardiac differentiation and maturation, as the heart is an electro-sensitive organ. Hernández et al. differentiated hiPSCs into cardiac cells by forming embryoid bodies (EBs). Electrical stimulation at 65 mV/mm or 200 mV/mm for 5 min significantly increased the percentage of beating EBs and the cardiac gene expression of ACTC1, TNNT2, MYH7, and MYL7 [73]. Creating dynamic bioreactor systems that facilitate the delivery of controlled electrical cues aid in the functionalization of cardiac constructs [74]. Wang et al. designed a multi-stimulation bioreactor, which was capable of delivering both mechanical (20% strain) and electrical (5 V, 1 Hz) stimulations to the bioengineered cardiac construct. Porcine decellularized myocardium was reseeded with rat mesenchymal stem cells differentiated into cardiac-like cells using 5-azacitidine. Results showed that combined stimulations have a synergic effect, promoting better cell repopulation in the myocardial scaffold [75]. In conclusion, the design of a bioreactor needs combining biochemical, biomechanical, and electrical controls for a balanced environment in cardiac tissue growth.

## 4. ECM Decellularization Methods

**Figure 3:**
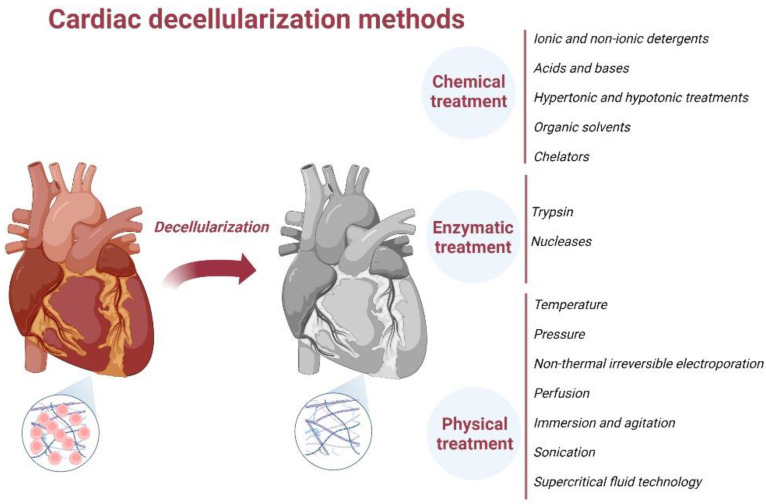
Cardiac decellularization. The decellularization removes all cellular components, creating a stable, biologically active scaffold. Different decellularization methods were used over time, including chemical, enzymatic, physical or combination. Created with BioRender.com.

Maintaining the balance between the clearance of cellular components and the retention of a comparable native ECM represents the primary goal of decellularization. Decellularization is based on several chemical, enzymatic or physical treatments (Figure 3). ECM structure is highly organ-specific as its components influence cell specialization and tissue function [76].

Each decellularization protocol produces a different impact on ECM structural proteins. Thus, the decellularization protocol must be chosen based on the tissue structure and function. Combined decellularization strategies may improve the process efficiency and limit the negative effects caused by using a single technique [77].

### 4.1. Chemical Treatment-Based Decellularization

#### 4.1.1. Ionic and Non-Ionic Detergents

Solutions containing detergents have been proven to be the most effective decellularization method. Among the commonly used agents, sodium dodecyl sulfate (SDS) and Triton X-100 are preferred [78]. Non-ionic detergents such as Triton X-100 are considered gentle detergents, maintaining the ultrastructure of the ECM but being less effective in removing cellular debris [11,77,79]. In 2008 Liao et al. demonstrated successful decellularization of the porcine aortic valve using 1% Triton X-100. This research underlines that the differences in tissue density and cellularity interact with decellularization effectiveness [80].

Ionic detergents such as SDS can completely disrupt cell membranes, decreasing collagen integrity, but it is very effective in cell removal [77,81]. As an aggressive reagent, studies have shown that concentrations superior to 1% induce collagen, elastin precipitation, and ECM denaturation [82,83]. Therefore, the results of previous research suggest applying multiple low-concentration washes with a short exposure period when using SDS protocols [84].

#### 4.1.2. Acids and Bases

The way of acid action is by solubilizing cytoplasmic elements, disrupting nucleic acids, and denaturing ECM proteins [85]. Peracetic acid (PAA) 0.3% treatment was used to decellularize and sterilize vascular scaffolds. In combination with DNase I, this approach proved patent for two weeks in allogeneic rat transplantation, followed by graft rupture because of the decreased thickness in the ECM [86]. Acids and bases are mainly used in combination with other decellularization agents. In previous work, Mendoza-Novelo et al. used a calcium oxide protocol for bovine pericardium decellularization. The stress relaxation of the ECM was lowered, and the GAG content reduction was more severe when compared with non-ionic detergents alone [87]. Chemical acid–base treatments are rarely used for cardiac tissue decellularization because they are frequently aggressive toward the proteins of the ECM.

#### 4.1.3. Hypertonic and Hypotonic Treatments

Hypertonic/Hypotonic solution treatment relies on the osmotic effect to remove cellular components from the cells. These solutions easily kill the cells by causing swelling or lysis [88,89]. Examination of porcine vessels that were exposed to a hypotonic solution (10 mM Trizma HCl, 5 mM EDTA) and hypertonic solution (50 mM Trizma HCl, 1 M NaCl, 10 mM EDTA) did not remove the cellular remnants, and no significant difference in DNA was detected between treatment and control [89]. Most previous research using this technique resulted in inadequate immunogenic conditions for implantation. However, the last attempts described an effective removal of DNA content, making the resulting constructs potentially functional bioartificial tissues [90]. Until now, no hypertonic/hypotonic solution protocol has been developed for whole heart decellularization.

#### 4.1.4. Organic Solvents

Chemical-based decellularization techniques using solvents should be used with caution because of the potential damage to the 3D microstructure and unsuccessful recellularization [91]. Ethanol or methanol are usually needed to remove residual DNA from tissue. Levy et al. showed that ethanol pretreatment of bioprosthetic heart valves alters the collagen structure, leading to unexpectedly severe leaflet calcification [92].

Chemical decellularization is a frequently used method for cardiac scaffold preparation and provides high efficiency in cell removal (Table 2).

### 4.2. Enzymatic Decellularization

#### 4.2.1. Trypsin

This enzyme is used in many decellularization protocols targeting the C-side bonds in lysine and arginine amino acids but disrupting the tissue microstructure when applied for too long. Trypsin is mainly used with detergents or enzymatic chelating agents (e.g., ethylenediaminetetraacetic acid—EDTA) [93,95,96]. A study published in 2013 by Merna et al. demonstrated that decellularization of whole porcine hearts using trypsin significantly decreased DNA while severely reducing the structure and mechanical integrity of the ECM [79]. Trypsin is rarely used as a single treatment for decellularization, but it is limited to an initial pretreatment step before decellularization with other agents [97,98]. Perfusion of murine hearts with a trypsin-EDTA solution for 20 min, followed by washing with a mixture of ionic and non-ionic detergents, resulted in acellular scaffolds with proper 3D architecture maintenance. The decellularized ECM was further repopulated with human iPS cell-derived multipotential cardiovascular progenitors (MCPs) [99].

#### 4.2.2. Nucleases

Nucleases (e.g., DNases and RNases) target intracellular contents and are usually used alongside other decellularization agents to be effective. Regarding heart valve decellularization, nucleases at low concentrations have been used after detergent treatment and they achieve an almost complete DNA removal. In addition, the morphology of the decellularized leaflets was preserved as well as type III collagen components of the basement membranes [100]. Wang et al. established a decellularization protocol for minced neonatal mouse hearts without using detergents but with a cocktail containing 250 U/mL DNase and 25 U/mL RNase. The bioactivity of the acellular ECM was analyzed in an in vivo model of myocardial infarction showing cardiac function and revascularization improvement [101]. Ramm et al. combined chemical decellularization with enzymatic treatment (PNGase F and DNase I) to remove N-linked glycans and residual DNA of porcine pulmonary heart valves (pPHV). Implantation of decellularized pPHV in sheep for six months proved excellent hemodynamic performance, with no increase in the mean valvular gradient or insufficiency [102].

Enzymes have been used as biological methods for decellularization with some adverse effects on the ECM, as labeled below (Table 3).

### 4.3. Physical Decellularization

#### 4.3.1. Temperature

Freeze–thaw processing works by forming intracellular ice crystals that disrupt cellular membranes causing cell lysis. After a freeze–thaw cycle, a subsequent process should be used to remove all the cellular remnants. Early in 2010, Lehr et al. demonstrated that a single freeze–thaw cycle could reduce the immunogenicity of ovine decellularized allograft pulmonary artery patches [103]. Snap freezing may cause certain disruptions of the ECM ultrastructure. Some researchers suggested using extracellular cryoprotectants (5% trehalose) to maintain the ECM molecular network without restraining the cell lysis [104]. Wainwright et al. published a protocol that started with freeze cycles, followed by enzymes and detergents, obtaining a completely decellularized porcine heart after ten hours. The exposure of the organ to a temperature of −80 °C (for at least 16 h) shortened the decellularization time, minimizing the damage to the ECM [15].

#### 4.3.2. Pressure

An effective decellularization method uses high-hydrostatic pressure (HHP) technology, disrupting the cells inside the tissue. Funamoto et al. described an HHP decellularization treatment (30 °C starting temperature, 65.3 MPa/min pressurization and depressurization rates) of porcine aortic blood vessels. Results showed no mechanical properties alteration and no cellular debris detection. After allogeneic transplantation of the decellularized tissue, cellular infiltration on the vessel wall could be observed, with no thrombus formation [105]. A more physiological decellularization method was described by placing the organ inside a pressurized pouch in an inverted orientation under controlled pressure (constant pressure of 120 mmHg measured at the aortic root), improving myocardial perfusion. Combined with chemical treatment, this method enhances the decellularization of non-transplantable human hearts [56].

#### 4.3.3. Non-Thermal Irreversible Electroporation (NTIRE)

This approach to tissue decellularization involves the formation of micropores in the cell membrane by applying microsecond electrical pulses. Therefore, cell homeostasis is lost, leading to cell death. The molecular mechanisms of cell death after NTIRE are still not precisely understood [106,107]. Researchers described in vivo and in vitro tissue and organ decellularization using NTIRE, causing irreversible cell damage while sparing the ECM [108,109]. Exciting data were obtained by Zager et al. in 2016 regarding irreversible electroporation protocols for in vivo beating heart model decellularization. Twenty-eight days of follow-up data reflect remodeling of the left ventricular myocardium following NTIRE [110].

#### 4.3.4. Perfusion

Perfusion decellularization is perhaps the most widely recognized method of removing cells. This decellularized route is preferred for whole organs. It offers the possibility of establishing a channel for circulating detergents through the intrinsic vascular system [111]. The efficiency of a decellularization perfusion protocol depends on various factors such as perfusion route, selected perfusate, perfusion parameters, and organ dimension [11,15]. Since the pioneering work of Ott et al. in 2008, perfusion decellularization protocols have started to build upon the concept. Inverted orientation of a porcine heart (perfusion pressure of 60 mmHg and −45° angled heart) during detergent perfusion guided superior cellular remnants outflow, lower deoxyribonucleic acid (DNA), and higher collagen and elastin content in the ECM, as well as better retention of the heart shape [17].

#### 4.3.5. Immersion and Agitation

This technique involves immersion of the organ in a decellularization solution, followed by shaking on a stir plate to facilitate the rupture, detachment, and removal of cellular components [112]. Protocols using this physical decellularization method have been described for numerous tissues, including cardiovascular tissue [18,113,114]. In 2014, Methe et al. described an alternative approach for whole porcine heart decellularization by serial perfusion with detergents (4% sodium deoxycholate followed by 1% Triton X-100) and immersion in a sterile beaker on an orbital agitator (at 37 °C). Further assessment of the ECM showed near complete removal of cellular components and a well-preserved 3D acellular scaffold with highly organized cytoskeletal elements [115].

#### 4.3.6. Sonication

Ultrasonic waves can disrupt the cellular membrane and release intracellular components. Cavitation formation during sonication caused by the ultrasonic waves facilitates the detergent-based decellularization process. Unfortunately, uncontrolled lower frequencies can damage tissue’s structure and mechanical properties [116,117]. Hazwani et al. increased the effectiveness of aortic tissue decellularization using a closed sonication system. Fresh porcine aortas were sonicated at 170 kHz of ultrasound frequency in 0.1% and 2% sodium dodecyl sulfate (SDS) for 10 h. Subsequently, the tissue was washed in phosphate-buffered saline solution (PBS) for 5 days to clear the residual detergent. Hematoxylin and eosin (H&E) staining confirmed the removal of all cells. The ECM surface maintained the fibrous collagen network and elastin fibers, the sonication treatment causing minor damage to the main elastin content. The ultrasonic waves did not significantly affect the biomechanical properties, only slightly increasing the stiffness and decreasing the residual force. The bioscaffolds after ultrasonic treatment were evaluated 1 and 5 weeks after subcutaneous implantation in rats, showing minimal inflammatory response [118]. Lin et al. used sonication-assisted decellularization to reduce the SDS exposure time of the human umbilical artery (HUA). The process was tested at 40 kHz, followed by a washing procedure. The bioscaffolds were implanted into rats as an abdominal aorta bridge and assessed by magnetic resonance angiography (MRA). The histological evaluation of sonication-treated HUA showed the removal of cellular components. The best results regarding ECM structure and mechanical properties preservation were obtained using the 204 W for 4 h protocol. This research showed that sonication-assisted decellularization (204 W for 4 h followed by 2% SDS treatment) is an effective decellularization method. Still, higher power or more extended duration treatment increases cavity formation and layer dissociation in the vessel wall [119].

#### 4.3.7. Supercritical Fluid Technology

Supercritical fluids (e.g., carbon dioxide) allow for simple and fast decellularization protocols. Supercritical carbon dioxide (scCO_2_) provides an advantage over conventional decellularization methods eliminating additional sterilization steps since scCO_2_ also acts as a bactericidal agent and viral inactivator [120]. Complete decellularization is usually not attained by only treatment with scCO_2_; therefore, an initial decellularization treatment with a chemical agent such as alcohol is required [121,122,123]. Topuz et al. evaluated a scCO_2_-assisted decellularization method, using pre-treatment with a hypotonic (10 mM Tris-HCl, pH 7.9) and hypertonic solution (1.5 M NaCl in 0.05 M Tris-HCl, pH 7.6). After the pre-treatment, the myocardium was decellularized for 1 h in a scCO_2_ reactor filled with 150 mL 70% (*v/v*) ethanol. Decellularized myocardium expressed a 60% reduction in residual DNA, with 64% of the GAG contents preserved compared to the original tissue [124]. Gafarova et al. performed a comparative analysis of three decellularization treatments of the ovine aortic root in alkaline (1 M NaOH + 0.8 M Na_2_SO_4_), alcohol (95% ethanol), or detergent solutions (0.5% SDS/0.5% sodium deoxycholate SDO) with scCO_2_-assisted processing. The control groups included untreated native samples (negative control) and detergent-only treated samples (positive control). The ethanol and alkali-based decellularization did not achieve satisfactory results for the aortic root in terms of effectiveness and preservation of ECM components [125]. In contrast, research conducted by Halfwerk et al. obtained efficient alkali-based scCO_2_-assisted decellularization of porcine and bovine pericardium without significantly affecting its mechanical and structural properties [126]. The SDS/SDO-based protocols as a preconditioning medium followed by scCO_2_-assisted processing revealed favorable effects with mechanical properties comparable to that of native tissue [125].

Physical treatments are typically used to complete chemical and enzymatic treatments and increase the decellularization effects (Table 4).

### 4.4. Combination of Chemical, Enzymatic and Physical Methods

Researchers have developed optimized decellularization protocols by combining different agents in order to obtain the perfect balance between cell removal, ECM composition, and properties maintenance. Numerous studies have been implemented on perfusion decellularization systems for the whole heart. This process generally involves passing a decellularization agent via the intrinsic heart vasculature, washing out the cellular components [11,127,128,129,130]. In previous work, we obtained an optimal decellularization protocol for rat hearts using a modified Langendorff experimental device (constant perfusion pressure of 80 mmHg) in the presence of an alternating rectangular electric field (20 kHz frequency and 100 mA amplitude, corresponding to 7.14 mA/cm^2^ current density) [131]. Akhyari et al. compared three previous protocols with a newly developed fourth protocol which implied the decellularization of whole murine hearts through coronary perfusion. Saponin, a mild detergent rarely used for decellularization, was combined with 1% SDS and 1% deoxycholic acid (DCA). The conclusion was that no single strategy was superior to the others, being difficult to achieve the optimal decellularized biological scaffold [132]. Lee et al. examined two novel retrograde decellularization methods for porcine hearts and combined chemical solutions (500 mM NaCl hypertonic solution, 20 mM NaCl hypotonic solution, and 1% SDS), demonstrating the importance of heart orientation in the process [17]. Decellularization protocols should be optimized to species source and organ age. The same protocol for porcine hearts proved ineffective when applied to the same size cadaveric human hearts [38,133].

Many researchers believe that SDS is the optimal treatment for cardiac tissue decellularization. However, studies have shown that SDS is as effective as it is aggressive to ECM depending on the concentration, leading to several mechanical dysfunctions [93]. Thus, in 2022 Al-Hejailan et al. published an article based on the optimization of SDS and sodium deoxycholate (SDO) to obtain acellular porcine heart scaffolds. The SDS protocol established a more efficient removal of cells, with a highly preserved ECM structure confirmed by GAG quantification and immunohistochemistry [94].

Each decellularization technique has advantages and disadvantages, which must be carefully considered before clinical application.

## 5. Quantification of Complete Decellularization

Researchers rely on several available methods to assess cell material removal efficiency. Confirmation of successful decellularization is required before in vivo implantation [134]. Decellularization aims to remove DNA while preserving collagen and GAGs, which are major components of the ECM, a potent modulator of cell behavior [15,99,135]. Macroscopic assessment of the decellularized cardiac scaffold revealed a translucent aspect with intact geometry and vasculature tree of a native heart [11,52,127,136]. The histological analysis mainly using hematoxylin and eosin (H&E), Masson’s Trichome, or Movat’s pentachrome stain could confirm nuclei’s absence post-decellularization and a well-preserved ECM with a high number of GAGs, collagen, and elastic fibers [11,16,19,129,132,137,138]. Scanning electron microscopy (SEM) is frequently used for observing the microstructure of decellularized cardiac tissue. This high-resolution imaging revealed an intact aortic wall, valve leaflets, and missing myofibers in the dECM [11,99]. Immunohistochemistry staining for elastin, fibronectin, laminin, hyaluronic acid, and heparan sulfate was performed by Methe et al. in 2014 to prove that the decellularization process did not distort the structure of ECM components [115]. Positive immunofluorescence for collagen type I and elastin indicated maintenance of the mechanical properties and elasticity of the acellular cardiac scaffold [139]. The acellular state of decellularized heart biopsies could be confirmed using DAPI (4′,6-diamidino-2-phenylindole, dihydrochloride) staining with no evidence of cell nuclei or residue of nuclear material [11,115,139,140]. DNA quantification is an effective technique to describe the removal of genetic material. The main concern on nucleic material is justified because DNA is directly linked to adverse host reactions [141]. Crapo et al. proposed the following minimal criteria to satisfy the intent of decellularization: <50 ng double-stranded DNA (dsDNA) per mg ECM dry weight, <200 bp DNA fragment length, and lack of visible nuclear remnants in a tissue section stained with DAPI or H&E [12]. Bruyneel et al. compared various methods that express the tissue composition of decellularized scaffolds, signaling ambiguity because of incorrect normalization. Consequently, they proposed alternative comparison strategies: normalization to initial and final wet weight and final dry weight [142]. Before any clinical application, an acellular scaffold undergoes a series of in vitro procedures to improve its ability to successfully graft and function [78].

## 6. Pre-Application Processing

After in vivo transplantation of a decellularized tissue, a biodegradation process occurs, affecting the scaffold’s mechanical strength and durability. In addition, acellular tissues following decellularization are softer due to the removal of cellular components. To overcome these limitations and to ensure a proper 3D network structure, ECM-derived scaffolds usually undergo a cross-linking treatment. Cross-linking agents are classified into physical, chemical, and natural agents, having different properties but also some unwanted side effects on the ECM. Natural cross-linking agents exhibit superiority in aspects of cytotoxicity and anti-calcification ability [143,144,145]. Wang et al. decellularized porcine ascending aorta segments and treated them with procyanidins (PC), a type of natural polyphenols with cross-linking ability. Cross-linking aortic elastin with PC proved to significantly inhibit calcification and minimize the potential immunogenicity of decellularized tissues after subcutaneous implantation [146]. Chang et al. investigated genipin, another natural cross-linking agent, and compared it with glutaraldehyde (GA), a well-known toxic chemical agent causing tissue calcification. Acellular bovine pericardium treated with genipin was implanted subcutaneously in a rat model and showed a lower inflammatory response and faster tissue regeneration rate [147]. Cross-linking decellularized heart valves with nordihydroguaiaretic acid (NDGA) showed no disruptions to the native histoarchitecture. Regarding the mechanical properties, NDGA cross-linking increased the mechanical strength, surpassing the GA-crosslinked heart valve scaffolds. Lastly, NDGA proved excellent cytocompatibility to valvular cells, being an effective cross-linking agent for cardiac tissue engineering [148].

In the field of tissue engineering progress, sterilization is a critical challenge researchers must overcome to move toward clinical application. Ideal sterilization of decellularized constructs provides removal of microorganisms while maintaining the physical and chemical properties and biological activity [149]. Unfortunately, besides achieving effective sterilization, some agents are known to modify the ECM ultrastructure and properties [81,120]. Sterilization methods for cardiovascular dECM mainly include gamma irradiation [150], ethylene oxide (EO) [151], peracetic acid (PAA) [152] and supercritical carbon dioxide (ScCO_2_) [153]. Helder et al. evaluated low-dose gamma irradiation as a sterilization method for decellularized porcine heart valves. Post-operative follow-up in a sheep model of pulmonary valve replacement (*n* = 3) showed significant structural and functional changes in the valve leaflets. The valves failed due to ineffective sterilization (causing bacterial endocarditis) from using 1500 Gy gamma irradiation or damage from using 3000 Gy gamma irradiation. The study concluded that gamma irradiation might not be ideal for sterilizing decellularized heart valves [150]. Zhao et al. obtained tissue-engineered blood vessels using decellularized ovine arterial scaffolds and autologous bone marrow-derived MSCs. The acellular constructs were sterilized with EO gas at room temperature. MSCs-seeded vascular grafts implanted into the arteries of sheep proved patent, anti-thrombogenic, and mechanically stable for 5 months in vivo [151]. A study published by Luo et al. used 3T3 murine fibroblasts and BHK baby hamster kidney cells to test the biocompatibility of acellular porcine pulmonary valves decontaminated in 0.1% PAA. In vitro biocompatibility studies indicated that the acellular scaffolds were not cytotoxic [152].

Antibiotic treatment alone is also used to obtain an aseptic state of the dECM. It inhibits the growth of bacteria and has no obvious negative effects on the dECM [154,155]. In 2018 Fidalgo et al. investigated the efficiency of a two-step sterilization strategy combining an antibiotics/antimycotic cocktail with PAA. This treatment provided aseptic scaffolds with preserved structural integrity and biocompatibility [156]. Factors including application purpose, physical and chemical properties of tissue, or time required are essential for selecting the best sterilization method [149].

Preserving cardiac decellularized scaffolds involves keeping them in phosphate-buffered saline solution (PBS) with antibiotics and antimycotics at 4 °C for a short period [21]. Long-term preservation strategies to much lower temperatures include conventional cryopreservation, vitrification, and freeze-drying. All preservation methods proved no collagen denaturation or loss of elastin and GAGs of decellularized bovine pericardial (DBP) scaffolds. However, cryopreservation significantly changed the biomechanical behavior of the DBP scaffolds, which might lead to graft dysfunction in vivo [157].

## 7. Application of dECM in Regenerative Medicine

Researchers engineer decellularized tissues, with or without recellularization, to augment or fabricate ventricular myocardium. Decellularized and recellularized scaffolds have the potential to substitute heart valves, to produce cardiac patches and blood vessel grafts, or ultimately to be used for whole heart transplantation (Table 5) [158,159].

A study by Wang et al. demonstrated that decellularized neonatal cardiac ECM could prevent widespread ventricular remodeling after injury using an in vivo acute myocardial infarction (MI) model. Decellularized neonatal mouse hearts (*n* = 30) were minced, producing a powder resuspended into a hydrogel. A single injection of neonatal mouse ECM (nmECM) into the injured ventricle improved cardiac function. Echocardiographic measurements at 6 weeks post-MI showed that nmECM reduced the enlargement of the end-diastolic area (EDA) and the end-systolic area (ESA) and slowed the left ventricle ejection fraction (LVEF) decline compared to saline and adult mouse ECM-treated mice (*n* = 3 for each group). MI-induced fibrosis and CD68+ population were reduced in nmECM-treated mice 6 weeks post-MI. NmECM increased human umbilical vein endothelial cells (HUVEC) migration and activity in hypoxic (2.5% O_2_) and nutrient-deprived media in vitro. Additionally, treatment with nmECM increased the density of CD31+ endothelial cells (EC) and αSMA+ smooth muscle cells in vivo [101]. Shah et al. examined the therapeutic outcome of decellularized porcine myocardium slices (dPMS) as an acellular cardiac patch for the infarcted area in a rat acute MI model. Assessment at four weeks after surgery showed that LVEF and wall contraction improved. Simultaneously, dPMS promoted neovascularization from infiltrated host cells after one week of transplantation [160]. Sarig et al. evaluated the regenerative capacity of decellularized porcine cardiac ECM patches (pcECM-P) when implanted in both acute and chronic MI models. The results revealed that decellularized porcine patches could prevent further deterioration and improve contractility and cardiac remodeling in rat hearts that underwent MI. Additionally, pcECM-P recruited progenitors that differentiated into CM-like cells, which self-organize muscle ‘fiber-like’ patterns [161]. Aortic valve replacement with decellularized allograft has seen positive outcomes when implanted into sheep. Echocardiography assessment revealed a normal appearance with mild insufficiency. Characterization of the explanted decellularized aortic valve (after three months and nine months) showed no signs of calcification, sclerosis, or rejection [164].

Researchers have investigated the efficacy of seeding acellular cardiac scaffolds with various stem cells. Studies showed that cardiac dECM delivers topographical and biological signals that regulate cell differentiation and maturation in tissue development [168,169]. An article published in 2016 by Wang et al. described obtaining functional engineered human cardiac patches using decellularized natural heart matrix and human iPSCs differentiated into the cardiovascular lineage. The engineered patches exhibited spontaneous contractions in vitro and, when implanted into rats, improved echocardiographic parameters such as LVEF [10]. Perea-Gil et al. compared engineered cardiac grafts based on decellularized scaffolds from porcine myocardium (myo) and human pericardium (per) and repopulated them with porcine adipose tissue mesenchymal stem cells (pATMSCs). Decellularized scaffolds maintained intrinsic organization and spatial 3D distribution of the native matrix fibrils. One-week post-recellularization, pericardial scaffolds expressed superior cell distribution with complete migration of pATMSCs throughout scaffold thickness compared to the myocardial scaffold. Macro and micromechanics were well-maintained following decellularization, but recellularized myocardium micromechanics was ∼2-fold stiffer. For preclinical tests, pigs (*n* = 74) were submitted to MI (8 animals were excluded due to death or post-operative infections) and distributed into five groups: control MI, Per/Myo-MI with cell-free scaffold implantation, and Per/Myo-ATMSCs enriched scaffold implantation. Implantation of the engineered cardiac grafts guided improvements in LVEF and/or left ventricular end-systolic volume (LVESV) and limited infarct size expansion [162].

A systematic review conducted by Porzionato et al. in 2018 exposed the benefits of using pericardial tissue in cardiac surgery, whether substituting cardiac valves or repairing the ventricular wall [170]. Decellularized pericardium has recently emerged as a promising scaffold because it has been demonstrated to overcome the limitations of xenogeneic pericardial tissue treated with glutaraldehyde (GA). GA cytotoxicity and its potential immunogenic reactivity represent critical limitations for long-term performance [171,172]. In a preclinical model of swine MI, Galvez-Monton et al. used decellularized human pericardium seeded with porcine adipose tissue-derived progenitor cells (pATPCs) as an engineered bioactive impedance graft (EBIG). Results showed that the recellularized pericardial constructs exhibited better cardiac contractility (assessed by LVEF) and reduced infarct size. The bioengineered scaffolds lowered the inflammatory response and maintained better collagen I/III ratio [163].

Several studies are also ongoing into the building of engineered cardiac valves. Dohmen et al. reported in 2007 that decellularization treatment of pulmonary allografts and xenografts seeded with autologous vascular endothelial cells resulted in successful tissue-engineered heart valve implantation in 23 patients. Mid-term follow-up using echocardiography and computer tomography showed excellent hemodynamic performance, smooth and pliable leaflets, without calcification [165]. In 2010 a retrospective, nonrandomized, multicenter cohort analysis compared the clinical outcome of patients (*n* = 342) receiving the first decellularized pulmonary human heart valve (CryoValve SG Pulmonary Valve—SGPV) cleared by the United States Food and Drug Administration (FDA) with conventionally processed valves (*n* = 1246). SGPVs were prepared using SynerGraft decellularization technology, implying hypotonic lysis and nuclease digestion of the cellular elements. SGPV provided a functional valve with suitable echocardiographic parameters (significantly lower pulmonary insufficiency grades in SGPV recipients), a reduced incidence of calcification (11% of the valve-related explantation, 0.3% of the total patients receiving SGPV), and a reduced incidence of endocarditis (*n* = 1) at 4-year follow-up. The study has some limitations, like patients’ age between the two cohorts and the lack of standard protocol for reporting echocardiographic data [166]. On the other hand, studies have reported the failure of decellularized xenogeneic heart valves, such as the Matrix P Plus valve (MP-V), when implanted into patients to reconstruct the right ventricular outflow tract (RVOT). The graft failure was related to the massive inflammatory reaction and fibrosis [173,174].

Besides building parts of the heart, whole bioengineered hearts are tested through transplantation. Taylor et al. implanted porcine decellularized hearts acutely (*n* = 9) and chronically (*n* = 2) in living recipients in a heterotopic position allowing the graft to be connected to the native heart. Hearts were decellularized using a combined protocol with hypertonic (500 mM NaCl) and hypotonic (20 mM NaCl) solution, 1% SDS, 0.01% PAA and a final wash with PBS. Short-term implantation (4–6 h) evaluated the ability of the decellularized heart to recruit the recipient’s cells when connected to its circulation. The recipient’s blood and endothelial cells populated the decellularized heart, evidenced by CD31 and von Willebrand factor (vWF) staining. Long-term implantation at 60 days promoted tissue formation with evidence of what appeared to be nascent muscles in the graft. Unfortunately, the study could not detect coronary circulation patency in chronic cases. The authors also suggested that endothelization of the scaffolds before heterotopic transplantation could minimize coagulation dysfunction [167]. Researchers hope bioengineering human-sized functional hearts becomes an achievable end goal within our lifetime. It has been more than ten years since the pioneering work of Ott et al., and since then, researchers have worked to scale up the model to human size and use stem cells from human lineages to recellularize the acellular scaffolds [175,176].

## 8. Challenges in Cardiac Tissue Engineering

Preserving the microarchitecture and composition of the ECM during decellularization requires optimal protocols that provide efficient cell removal with minimal disruption [94]. The balance between effective cell removal and preservation of structural, biochemical, and biomechanical properties remains challenging for obtaining a cardiac dECM scaffold that will minimize the immunogenicity after implantation and that will reach the desired cell-ECM interaction [177]. Inefficient decellularization with remnant genetic materials could trigger immune-mediated rejection after in vivo implantation [112]. The four decellularization protocols compared by Akhyari et al. for the whole heart summarized perfectly the imbalance between cell removal and preservation of ECM. The treatments that resulted in better preservation of ECM proteins could not efficiently remove residual DNA. Correspondingly, when significant removal of cell debris was achieved, the cardiac dECM lost crucial components, like elastin and collagen IV [132]. Similarly, human cardiac thin slices (350 μm thick) were decellularized using five protocols, which proved substantially different. Only three protocols were established effective in producing acellular scaffolds. However, the cardiac dECM varied in architecture and ability to support engraftment, survival, and differentiation of cells in vitro [36]. A practical approach uses strong decellularization agents (1% SDS and 1% Triton X-100) for a short time, followed by consecutive PBS rinses, with an adequate balance of DNA removal and maintenance of ECM [178,179,180]. For a better decellularization strategy, the protocols should be standardized for cardiac tissue to obtain an ideal dECM for clinical application.

Another challenge for cardiac tissue engineering implies the recellularization of cardiac dECM. The most commonly seeding approach includes perfusion of stem cells, direct injection, or a combination of the two [11,32,99,181,182]. Unfortunately, no seeding strategy has proven optimal. For example, perfusion of decellularized porcine whole heart with human umbilical vein endothelial cells (HUVECs) via aorta followed by intramural injections reported incomplete recellularization due to inhomogeneous distribution and loss of cell during perfusion [16]. The static cell seeding strategy exhibited high cell density near the surface with limited infiltration in the core of the cardiac dECM. The bilateral cell seeding method with human MSCs (hMSCs) and rat adipose-derived stem cells (rASCs) had a higher efficiency on decellularized porcine myocardium slices [183,184].

In addition to the seeding strategy, determining the number of cells needed is another issue researchers must overcome. Isolation and expansion of large quantities of cardiomyocytes required to repopulate a human-sized decellularized heart are challenging. An adult human heart contains approximately four billion highly specialized cardiomyocytes [185]. The discovery of iPSCs and their ability to differentiate into beating cardiomyocytes has offered an unprecedented opportunity in cardiovascular regenerative medicine [186]. The ability to derive iPSCs from human adult cells is a potential solution to the large number of human cells needed for cardiac tissue engineering. Still, innovative methods for efficient expansion of iPSC-cardiomyocytes (CMs) are needed to improve the scalability of tissue engineering models and accelerate their clinical application [45]. Differentiation of CMs from human iPSCs critically depends upon regulating of the Wnt/β-catenin signaling pathway [187]. Wang et al. proved that XAV939, a small molecule inhibitor of Wnt/β-catenin signaling, can induce cardiomyogenesis in mouse embryonic stem cells. The formation of cardiomyocytes was confirmed using immunostaining (positive for α-actinin and cardiac troponin-T) and quantitative real-time PCR (increased Myh6 gene expression and cardiac marker Nkx2.5 expression). Additionally, Western blotting with cardiac troponin-T antibody showed a higher level in the XAV939-treated group compared to the control [188]. Tsoi et al. recently published a study that analyzes the WNT signaling pathway to generate highly pure hPSC-CM cultures. Cardiac differentiation efficiency was compared using three induction schedules and two combinations of WNT inhibitors (CHIR-99021, respectively IWP-2/IWR-1-endo/XAV939). The results showed that the temporal control of the WNT signaling pathway could regulate the maturation stage of derived hPSC-CMs [189].

Successful approaches for the in vitro generation of functional engineered cardiac tissues need a proper combination of cells, scaffolds, and cardiac-like biochemical and biophysical signals. Organ culturing and stimulation requires growing it in a perfusion bioreactor that delivers a nutrient-rich environment. Such a system provides a sterile environment while allowing for modifications in stimuli to be made [190]. Massai et al. developed a bioreactor platform for in vitro mechanical stimulation of engineered cardiac tissue (ECT). The constructs were exposed to four days of uniaxial cyclic stretch (sinusoidal waveform, 10% strain, 1 Hz) within the bioreactor. Exposed to electrical pacing on day 9, dynamically cultured ECT was responsive, exhibiting synchronous and regular contractile activity [191]. Hochman-Mendez et al. reseeded decellularized rabbit hearts with cardiac cells (CCs) and endothelial cells (ECs) differentiated from hiPSCs and generated a completely anatomically restored left ventricular (LV) wall. Whole heart recellularization experiments were performed using an adapted rabbit heart Langendorff perfusion system. Cells were delivered by infusion (100 × 10^6^ ECs) and injection (842.0 ± 363 million CCs) into the LV-free wall. Recellularized LV wall expressed spontaneous electrical activity, responded to chronotropic drug administration, and maintained vessel patency [192]. The complex natural microenvironment of the cardiac tissue dictates the development of customized bioreactors that meet specific clinical requirements. The design of a bioreactor needs standardized protocols (e.g., adjustment of the electrical stimulation parameters and better reproduction of physiological conditions such as the Frank-Starling response) [69]. Future clinical applications must address the ability to integrate engineered products with other organs and systems to simulate physiology acutely [193].

Stem cells combined with tissue engineering are believed to complement the spectrum of medical care in the future, with broad application prospects in tissue regeneration and organ transplantation. Therefore, researchers continuously investigate the post-transplantation risk of tumorigenesis, especially for ESCs and iPSCs [194]. Inefficient differentiation of iPSCs into cardiomyocytes increases the tumorigenic risk secondary to contamination with undifferentiated cells and non-cardiomyocytes. Several reports described different methods for removing those cells and preventing teratoma formation [195,196,197]. Patient safety is a primary focus in phase 1 clinical trials. Consequently, researchers have to evaluate the factors influencing progenitor stem cells’ tumor formation before clinical implementation.

The goal of creating functional whole human hearts has not yet been achieved. However, researchers are increasingly focusing on creating cardiac patches derived from dECM that may be just as effective in repairing damaged myocardium. A summary of these studies is seen in Table 5. While much progress has been made in recent years, important challenges still remain. Studies showed that implantation of cardiac patches might induce arrhythmia and cause worsening cardiac function [198,199]. Xenografts are used more frequently for cardiovascular applications compared to human tissue. Implantation of xenografts can raise immune reaction concerns. Most recent studies using in vivo animal models of dECM therapy saw little immune response with implanted xenogeneic scaffolds [163,200]. Further research and surgical risk assessment are required before translation into patients.

Even though the ability of engineered functional human-sized heart constructs remains limited, ongoing efforts are likely to open new solutions in cardiovascular research.

## 9. Concluding Remarks

Cardiac decellularized ECM-based research is far from its maximum potential. Because the heart is unlike other organs, there have been challenges regarding in vivo transplantation of engineered hearts. A genuinely functional bioengineered heart implies perfect synergy between electrical, mechanical, and physiological mechanisms. Heterotopic transplantation of whole human-size hearts in living recipients with evidence of cardiac tissue formation within the graft brings the idea of creating functional tissue-engineering organs one step forwards [167,201]. The therapeutic potential of regenerative medicine stands to revolutionize the treatment of cardiac valve replacement, ischemic heart disease, or end-stage heart failure, bringing the field closer to meaningful clinical translation.

## Figures and Tables

**Table 1:** Comparison between native tissue-derived dECM versus cultured cell-derived dECM.

	Native Tissue-Derived dECM	Cultured Cell-Derived dECM
Advantages	- remarkable similarity to the original tissue in composition and mechanical properties - dynamic microenvironment for stem cell behavior	- possible for large-scale in vitro study - lower immunogenicity - ease of obtaining models of small tissue regions - minimize disease transmission by screening ECM-synthesizing cells
Disadvantages	- shortage of donors - higher immune response - risk of pathogen transmission using decellularized xenografts - difficult for large-scale in vitro study	- poorer mechanical properties to native ECM (unsuitable for certain applications) - challenging to prepare at a large-scale (impossible to obtain sufficient numbers of primary cells)

**Table 2:** Different chemical methods used for cardiac tissue decellularization.

Chemical Decellularization Techniques	Mechanism	General Disadvantages	Study Findings
**Ionic detergents (SDS)**	breaks non-covalent bonds	aggressive treatment cytotoxic; it requires vigorous rinsing	no intact cells or nuclei were detected in 1% SDS-treated rat hearts for 12 h, with preserved fiber composition and orientation [11] perfusion of porcine hearts with 4% SDS for 12 h lacked intracellular components but retained specific collagen fibers, proteoglycan, elastin, and mechanical integrity [16] decellularization of porcine hearts by repeated washing with 0.5% SDS resulted in 98% DNA removal with only 6 h of detergent exposure [18] 9 h treatment with 0.5% SDS of human left ventricular myocardium showed a pronounced reduction of major matrix components compared to the 3-step protocol (2 h lysis, 6 h 0.5% SDS, and 3 h FBS) [21] 24 h of 0.1% SDS treatment of porcine valve conduits was reported to be effective in cell removal but susceptible to recellularization with human cells [83] low SDS concentration for a limited time (0.5% SDS, 5.5 h) delivered acellular heart constructs (13.1 ± 5.8 ng/mg residual DNA) with maintained cytocompatibility (reseeded with human bone marrow-MSCs) [84] porcine aortic and pulmonary roots treated with different concentrations of SDS removed cells completely but caused strong structural alterations [93] an optimized 1% SDS-based decellularization protocol obtained acellular cardiac scaffolds with applicability to generate vascularized cardiac patches [94]
**Non-ionic detergents (Triton X-100)**	solubilizes cell membranes, disrupting lipid-lipid and lipid-protein connections	less effective in removing cellular debris	rat hearts treated with Triton X-100 for 12 h showed incomplete decellularization [11] decellularization of porcine hearts with 3% Triton X-100 resulted in incomplete decellularization with only 40% DNA removal [79] 48 h treatment with 5% Triton X-100 damaged the tissue architecture of the human myocardium but was not sufficient to remove cellular material [21]
**Acids and bases**	solubilize cytoplasmic elements, disrupting nucleic acids	frequently aggressive toward the proteins of the ECM mainly used in combination with other decellularization agents PAA increases ECM stiffness	submillimeter diameter vascular scaffolds decellularized with 0.3% PAA proved patent in rat allogeneic transplantation model for 2 weeks, followed by graft rupture [86] bovine pericardium pretreatment by reversible alkaline swelling (RAS) produced a severe reduction in GAGs and stress relaxation ratios [87]
**Hypertonic and hypotonic treatments**	induce cell lysis by osmotic shock	inadequate cellular removal ineffective for whole organ decellularization	hyper/hypotonic treatment alone for 72 h of human myocardium damaged the ECM and showed incomplete decellularization [21]
**Organic solvents (ethanol)**	dehydrates and lyses cells	potential damage to the ECM microstructure	ethanol pretreatment of bioprosthetic heart valves led to unexpectedly cuspal calcification [92]

**Table 3:** Different enzymatic methods used for cardiac tissue decellularization.

Enzymatic Decellularization Techniques	Mechanism	General Disadvantages	Study Findings
**Trypsin**	cleaves peptide bonds on the C-side of lysine and arginine	limited to an initial pretreatment step before decellularization with other agents insufficient for decellularization alone (used with EDTA)	decellularization of porcine hearts with 0.02% Trypsin resulted in incomplete decellularization with only 59% DNA removal [79] 0.5% Trypsin/0.2% EDTA solution significantly affected the flexural behavior of aortic valve leaflets, which displayed a looser ECM network [80] porcine valve conduits treated with 0.1% Trypsin/0.02% EDTA for 48 h presented incomplete cell removal, known to cause acute immunogenic response and early graft failure [83] digestion for 24 h of the bovine pericardium with 1% Trypsin/0.02% EDTA/RNase A/DNase I achieved efficient decellularization but severe structural destruction and changed mechanical property of the ECM [95] combined treatment of bovine pericardium with 0.25%Trypsin-EDTA/3% TritonX-100/4% DCA/0.1% PAA exhibited a highly distorted and damaged collagen matrix (unstable bioprosthetic scaffold) [97]
**Nucleases**	cleaves nucleotide bonds	needs other decellularization agents to be effective	post-treatment with nucleases (DNase, RNase) improved the removal of all residual components (98% of DNA content) [100] post-treatment after freeze–thawing with a cocktail of DNase I/ RNase produced acellular cardiac ECM used for ventricular remodeling in an in vivo model of acute MI [101] post-treatment with DNase I and PNGase F was used to remove residual nucleic acids and to cleave off N-linked glycans from ECM proteins [102]

**Table 4:** Different physical methods used for cardiac tissue decellularization.

Physical Decellularization Techniques	Mechanism	General Disadvantages	Study Findings
**Temperature**	crystals created in the freezing process disrupt cellular membranes causing cell lysis	may cause certain disruptions of the ECM ultrastructure ineffective at removing cells and genetic material, therefore used in combination with enzymes and detergents	pre-treatment of adult porcine hearts with low temperature (−80 °C for at least 16 h) assisted in cell lysis [15] a single freeze–thaw cycle could reduce adverse immune responses such as leukocyte infiltration of decellularized vascular allografts [103] extracellular cryoprotectants (5% trehalose) prevented freeze–thaw cycles to cause certain disruptions of the ECM ultrastructure [104]
**Pressure**	applying pressure destroys the cellular membrane	can damage the ECM components	HHP treatment showed excellent decellularization efficiency of porcine aortic blood vessels; an allogenic transplantation study showed that the acellular scaffolds reduced the host immune response, endured the arterial blood pressure, with no clot formation on the luminal surface [105] creating pressure gradients across the aortic valve to keep it closed improved coronary perfusion efficiency of SDS and provided a whole decellularized human heart [56]
**Non-thermal irreversible electroporation (NTIRE)**	formation of micropores in the cell membrane leads to cell death	electrical field oscillation can disrupt ECM	NTIRE proved to be safe and efficient for in vivo myocardial decellularization (it might become a viable means for scaffold creation via organ decellularization) [110]
**Perfusion**	it establishes a channel for circulating detergents through the intrinsic vascular system of the organs	tissues without innate vasculature cannot be decellularized	decellularization by coronary perfusion with 1% SDS of cadaveric rat hearts preserved the underlying ECM and maintained intact the vascular architecture and chambers geometry [11,127] decellularized cardiac ECM decellularized using a combination of enzymatic and chemical treatments via pulsatile retrograde aortic perfusion supported the formation of organized chicken cardiomyocyte sarcomere structure in vitro [15] efficient decellularization of heart-lung blocs perfused via the ascending aorta as well as via the trachea with 1% SDS could be the first step on the pathway to creating bioengineered transplantable heart-lung scaffolds [128] pressure-controlled perfusion decellularization enables whole-organ tissue engineering at a clinically relevant scale [111,129]
**Immersion and agitation**	causes cell lysis, facilitates chemical exposure and cellular components removal	severe stirring can damage the ECM needs complementary treatments to assess effective decellularization	immersion of porcine hearts in a decellularization chamber using a modified Langendorff Radnoti system produced acellular whole heart scaffolds [18,115] detergent-based decellularization of porcine pulmonary valves under continuous shaking conditions for 24 h delivered proper dECM for efficient recellularization with human endothelial cells [113]
**Sonication**	ultrasonic waves can disrupt the cellular membrane and release intracellular components	uncontrolled lower frequencies can damage tissue’s structure and mechanical properties	sonication treatment significantly influenced the detergent-based decellularization efficiency of thick tissues (porcine aortic wall) compared to conventional ways of shaking [116,117] decellularized porcine aortic scaffolds using a closed sonication system (170 kHz) in 0.1% and 2% SDS showed a minimal inflammatory response after subcutaneous implantation in a rat model [118] sonication-assisted decellularization provided an acellular vascular scaffold with in vitro cytocompatibility and in vivo biocompatibility in a rat abdominal aorta implantation model [119]
**Supercritical fluid technology**	facilitates chemical exposure, leading to cell removal	initial pretreatment with chemical agents is required	hybrid decellularization with chemical agents and scCO_2_ offered significantly reduced treatment times [121,122,123,124,125] scCO_2_ was found efficient in providing 100% sterility of the porcine decellularized aortic valves [120]

**Table 5:** Application of cardiac dECM in regenerative medicine.

dECM Source	Formulation	Animal/Human Model	In Vitro Recellularization	In Vivo Implantation	References
Neonatal mouse heart	Injectable hydrogel	Mouse MI model	Seeding of HUVEC	Injection of decellularized nmECM hydrogel in the injured ventricle	[101]
Porcine myocardium slice	Acellular cardiac patch	Rat MI model	-	Implantation of the acellular patch on the infarcted myocardium	[160]
Porcine myocardium slice	Acellular cardiac patch	Rat MI model	-	Implantation of the acellular patch on the infarcted myocardium	[161]
Porcine SIS-ECM	Human CFs enriched collagen-acellular scaffold	Rat MI model/Patient diagnosed with MI within 4 weeks requiring CABG	Seeding of human CFs	Implantation of the bioscaffold on the infarcted myocardium	[35]
Rat CF-ECM	3D engineered cardiac patch	Mouse MI model	Seeding of hEMSCs	Implantation of the engineered patch to the epicardial surface of the MI area	[65]
Rat heart	TEMS	Rat MI model	-	Epicardial implantation of TEMS	[66]
Rat heart	3D engineered cardiac patch	Rat MI model	Seeding of hiPSCs differentiated into the cardiovascular lineage	Implantation of the engineered human cardiac patch on top of the infarcted area	[10]
Porcine myocardium/human pericardium	Acellular per/myo scaffold; per/myo-pATMSCs enriched scaffold	Pig MI model	Seeding of pATMSCs	Implantation of the engineered cardiac grafts on top of the infarcted area	[162]
Human pericardium	pATPCs enriched acellular human pericardium	Swine MI model	Seeding of pATPCs	Implantation of the repopulated scaffolds on the ischemic myocardium	[163]
Ovine aortic valve conduit	Aortic root	Juvenile sheep	-	Orthotopic replacement of the aortic valve with decellularized allograft	[164]
Ovine carotid artery	Tissue-engineered vascular conduits	Sheep	Seeding of autologous MSCs differentiated into ECs-like cells and SMCs-like cells	Implantation of tissue-engineered blood vessels into the carotid artery	[151]
Porcine coronary artery	Tissue-engineered vascular patch	Rat aorta patch repair model	Seeding of rat ASCs	Implantation of decellularized arterial scaffold patch either with or without ASCs	[48]
Ovine pulmonary artery	Allogeneic vascular patch	Ovine artery patch repair	-	Implantation of decellularized arterial scaffold patch into the descending thoracic aorta	[103]
Porcine pulmonary heart valve	Xenogeneic valve prosthesis	Ovine model	-	Orthotopic implantation	[102]
Human and porcine pulmonary valve	Valve prosthesis	Patient with aortic valve lesions	Seeding of autologous vascular endothelial cells	Ross procedure (the diseased aortic valve is replaced with the pulmonary valve)	[165]
Deceased human donor	Allogeneic valve prosthesis	Patient with valve lesions	-	Ross procedure and RVOT reconstruction	[166]
Porcine heart	Whole organ	Pig and calf	-	Implantation of decellularized heart in the living recipient in a heterotopic position	[167]

MI—myocardial infarction; HUVEC—human umbilical vein endothelial cells; nmECM- neonatal mouse ECM; SIS-ECM—small intestinal submucosal extracellular matrix; CABG—coronary artery bypass graft; CFs—cardiac fibroblasts; CF-ECM—cardiac fibroblast derived extracellular matrix; hEMSCs—human embryonic stem cell derived mesenchymal stromal cells; TEMS—tissue engineered myocardial sleeve; hiPSCs—human induced pluripotent stem cells; pATMSCs—porcine adipose tissue mesenchymal stem cells; pATPCs—porcine adipose tissue-derived progenitor cells; ECs—endothelial cells; SMCs—smooth muscle cells; MSCs—mesenchymal stem cells; ASCs—adipose stem cells; RVOT—right ventricular outflow tract.

## Abbreviations

ECM—extracellular matrix; dECM—decellularized extracellular matrix; CMs—cardiomyocytes; 3D—three-dimensional; ESCs—embryonic stem cells; MSCs—mesenchymal stem cells; iPSCs—induced pluripotent stem cells; vSMCs—vascular smooth muscle cells; ECs—endothelial cells; bFGF—basic fibroblast growth factor; VEGF—vascular endothelial growth factor; CPCs—cardiac progenitor cells; HUVEC—human umbilical vein endothelial cells; GAGs—glycosaminoglycans; ASCs—adipose-derived stem cells; hDFs—human dermal fibroblasts; CFs—cardiac fibroblasts; hEMSCs—human embryonic stem cell-derived mesenchymal stromal cells; HHP—high-hydrostatic pressure; NTIRE—Non-thermal irreversible electroporation; DNA—deoxyribonucleic acid; SDS—sodium dodecyl sulfate; HUA—human umbilical artery; MRA—magnetic resonance angiography; PAA—peracetic acid; EDTA—ethylenediaminetetraacetic acid; MCPs—multipotential cardiovascular progenitors; pPHV—porcine pulmonary heart valves; DCA—deoxycholic acid; SDO—sodium deoxycholate; H&E—hematoxylin and eosin; SEM—scanning electron microscopy; DAPI—4′,6-diamidino-2-phenylindole, dihydrochloride; PC—procyanidins; GA—glutaraldehyde; NDGA—nordihydroguaiaretic acid; EO—ethylene oxide; ScCO_2_—supercritical carbon dioxide; PBS—phosphate-buffered saline solution; DBP—decellularized bovine pericardium; MI—myocardial infarction; nmECM—neonatal mouse ECM; EDA—end-diastolic area; ESA—end sistolic area; dPMS—decellularized porcine myocardium slices; LVEF—left ventricular ejection fraction; pcECM-P—porcine cardiac ECM patches; ATMSCs—adipose tissue mesenchymal stem cells; LVESV—left ventricular end-systolic volume; pATPCs—porcine adipose tissue-derived progenitor cells; EBIG—engineered bioactive impedance graft; SGPV—CryoValve SG Pulmonary Valve; FDA—Food and Drug Administration; MP-V—Matrix P Plus valve; RVOT—right ventricular outflow tract; vWF—von Willebrand factor; CCs—cardiac cells; LV wall—left ventricular wall.
